# Prostate cancer risk variants of the HOXB genetic locus

**DOI:** 10.1038/s41598-021-89399-7

**Published:** 2021-05-31

**Authors:** William D. Dupont, Joan P. Breyer, Spenser H. Johnson, W. Dale Plummer, Jeffrey R. Smith

**Affiliations:** 1 Department of Biostatistics, Vanderbilt-Ingram Cancer Center, Vanderbilt University Medical Center, Nashville, TN USA; 2 Department of Medicine, Vanderbilt-Ingram Cancer Center, Vanderbilt Genetics Institute, Vanderbilt University Medical Center, Nashville, TN USA; 3 Medical Research Service, Tennessee Valley Healthcare System, Veterans Health Administration, Nashville, TN USA; 4 Division of Genetic Medicine, Department of Medicine, Vanderbilt-Ingram Cancer Center, Vanderbilt University Medical Center, 507 Light Hall, 2215 Garland Avenue, Nashville, TN 37232 USA

**Keywords:** Cancer genetics, Prostate cancer, Predictive markers

## Abstract

The G84E germline mutation of *HOXB13* predisposes to prostate cancer and is clinically tested for familial cancer care. We investigated the HOXB locus to define a potentially broader contribution to prostate cancer heritability. We sought HOXB locus germline variants altering prostate cancer risk in three European-ancestry case–control study populations (combined 7812 cases and 5047 controls): the International Consortium for Prostate Cancer Genetics Study; the Nashville Familial Prostate Cancer Study; and the Prostate, Lung, Colorectal, and Ovarian Cancer Screening Trial. Multiple rare genetic variants had concordant and strong risk effects in these study populations and exceeded genome-wide significance. Independent risk signals were best detected by sentinel variants rs559612720 within *SKAP1* (OR = 8.1, *P* = 2E−9) and rs138213197 (G84E) within *HOXB13* (OR = 5.6, *P* = 2E−11), separated by 567 kb. Half of carriers inherited both risk alleles, while others inherited either alone. Under mutual adjustment, the variants separately carried 3.6- and 3.1-fold risk, respectively, while joint inheritance carried 11.3-fold risk. These risks were further accentuated among men meeting criteria for hereditary prostate cancer, and further still for those with early-onset or aggressive disease. Among hereditary prostate cancer cases diagnosed under age 60 and with aggressive disease, joint inheritance carried a risk of OR = 27.7 relative to controls, *P* = 2E−8. The HOXB sentinel variant pair more fully captured genetic risk for prostate cancer within the study populations than either variant alone.

## Introduction

Risk of prostate cancer is greatly elevated by the inheritance of a known germline mutation of *HOXB13* in men of European ancestry^1–10^. Genetic screening of cancer-prone families for the *HOXB13* G84E germline mutation is now encompassed by National Comprehensive Cancer Network (NCCN) Guidelines. A family with three or more affected first- or second-degree relatives meets criteria for hereditary prostate cancer. An early age of diagnosis is a recognized clinical facet of hereditary prostate cancer, observed in some although not all such families^11,12^. The HOXB13 transcription factor interacts with the androgen receptor to jointly regulate gene expression in the prostate. The corresponding network of activated genes is reprogrammed with transformation to prostate adenocarcinoma^13^. The eventual emergence of aggressive, castration-resistant prostate cancer can also be driven by HOXB13^14,15^. While additional missense variants of *HOXB13* have been observed, only G84E has been established to be associated with prostate cancer risk among men of European ancestry.

We investigated genetic variation of the chromosome 17q21 locus encompassing the HOXB cluster^16^ and neighboring genes to assess potential further contribution of regional genetic variants to prostate cancer risk. We evaluated three independent case–control study populations of European ancestry to replicate observations. Two of these study populations employed the familial case–control study design: the International Consortium for Prostate Cancer Genetics (ICPCG)^17^, and the Nashville Familial Prostate Cancer Study (NFPCS)^1,18,19^. This design uses family history as an index of genetic burden to improve power to detect infrequent and stronger effect variants. We also included a third case–control study population, that of the Prostate, Lung, Colorectal, and Ovarian (PLCO) screening trial^20^ (unselected for family history) to further evaluate observations made within the ICPCG and NFPCS. The combined total encompassed 7812 cases and 5047 controls. Array-genotyped and imputed variant data of these subjects enabled our identification of a series of novel genetic variants across the broader HOXB locus with strong risk effects, including the known *HOXB13* G84E mutation.

## Results

### Identification of HOXB locus variants predisposing to prostate cancer

**Figure 1:**
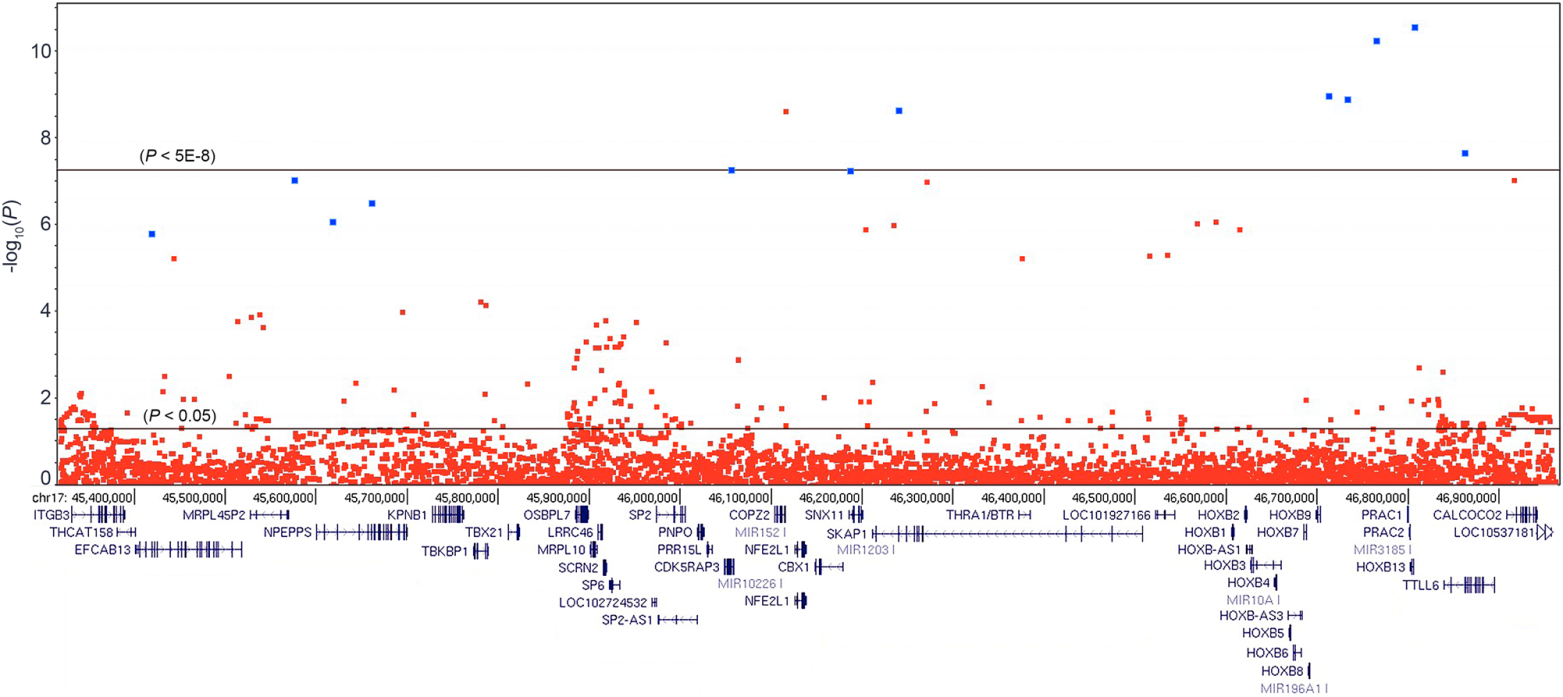
Association of genetic variants of the HOXB locus with prostate cancer in combined ICPCG, NFPCS, and PLCO subjects. Association tests for 5517 HOXB locus variants within subjects of the combined study populations are positioned along the X-axis (chr17: 45,316,626–46,960,760 genomic interval (GRCh37/hg19)), illustrating − log_10_ *P* values on the Y-axis. Horizontal black lines correspond to the genome-wide significance threshold of *P* = 5E−8 and to *P* = 0.05. Each data point depicts the result of a multiplicative logistic regression model (additive genetic model), with two-sided significance assessed using Wald tests. Variants depicted in blue are those that were also nominally significant in all three study populations separately (see Table 2). At bottom is a UCSC map of regional genes. Association results for each separate study population as well as for the combined study populations are given in Supplementary Table S1.

Multiple variants spanning the HOXB locus were nominally associated with prostate cancer with strong risk effects in all three study populations. We observed that in the familial case–control study populations of both the ICPCG and NFPCS (summarized in Table 1), the genomic interval between 17:45,416,600 and 17:46,860,777 (a 1.4 Mb interval of GRCh37/hg19) harbored numerous associated variants: 507 variants were nominally significantly associated with prostate cancer in ICPCG data, and 270 variants were associated in NFPCS data (see Supplementary Table S1). A shared overlap set of 69 variants were nominally significant and of a concordant direction of effect in both study populations; twelve of these variants had marked risk effects (Table 2). We comparatively evaluated data of the screen-detected PLCO study population in which cases were unselected for a family history of prostate cancer. Each of the twelve variants were also concordantly associated with prostate cancer in the PLCO with prominent, though less marked risk effects (Table 2). Carrier frequencies for these variants among the collective cases ranged from 1.1 to 2.6%. Figure 1 presents association results of the HOXB interval for subjects of all three studies combined. Variants depicted in blue were those that were nominally significant within each of the three study populations separately. Note that one of eight variants of Fig. 1 reaching genome-wide significance had replicated in only two of the three study populations (rs554574584, designated in red), with OR = 3.2 (*P* = 0.09) in the NFPCS. For each of the genome-wide significant variants, ICPCG and PLCO genotypic data had been imputed from array data (imputation *R*^2^ range 0.80 to 0.99; rs138467395 was an exception, genotyped in the ICPCG). We genotyped each of the seven variants of Table 2 reaching genome wide significance by custom assays in the NFPCS, confirming their associations. Strongest risk effects within the hereditary prostate cancer case subset of the NFPCS were observed for rs559612720 (*P* = 0.0096, OR = 16.2) and rs138213197 (*HOXB13* G84E, *P* = 0.0025, OR = 10.8). The rs559612720 mutation was carried by 2.4% and the rs138213197 mutation was carried by 3.4% of the combined ICPCG and NFPCS hereditary prostate cancer cases.

**Table 1:** Study populations.

	ICPCG^a^			PLCO^b^		NFPCS^c^			
	Control	Case		Control	Case	Control	Case		
Affected in pedigree	nr	≥ 3	2	nr	nr	0	≥ 3	2	1
European ancestry, count	1383	2505	2	2841	4599	823	331	344	31
Aggressive		56%	50%		nr		22%	43%	48%
Mean age at Dx or screen	nr	60	54	68	69	63	60	56	46
< 60 years	nr	45%	50%	8%	6%	43%	48%	67%	97%
≥ 60 years	nr	55%	50%	92%	94%	57%	52%	33%	3%

Dx, diagnosis; nr, not recorded.

^a^Subjects of 12 aggregated studies of the International Consortium for Prostate Cancer Genetics (ICPCG, dbGaP phs000733.v1.p1).

^b^Subjects of the Prostate, Lung, Colorectal, and Ovarian (PLCO) cancer screening trial's Prostate Cancer Genome-Wide Association Study for Uncommon Susceptibility Loci (PEGASUS, dbGaP phs000882.v1.p1).

^c^Subjects of the Nashville Familial Prostate Cancer Study (NFPCS). All subjects are genetically independent and of European ancestry. Aggressive is defined: ≥ pT3, or N1, or M1, or Gleason ≥ 8, or PSA ≥ 20 ng/ml, or lethal prostate cancer.

**Table 2:** Prostate cancer risk alleles of the HOXB locus.

Pairwise LD	Variant	chr:position	ICPCG		PLCO		NFPCS		Combined studies			
									OR	*P*	Carrier freq.	
			OR	*P*	OR	*P*	OR	*P*			Control	Case
	rs190859858_C	17:45416600	4.2	2.0E−04	2.0	0.036	4.6	0.019	3.0	1.5E−06	0.005	0.014
	rs559798379_C	17:45574038	4.1	1.0E−04	2.3	3.1E−03	3.4	0.039	3.0	8.8E−08	0.006	0.017
	rs369461501_T	17:45615944	4.2	1.6E−04	2.1	0.019	4.3	0.027	3.1	7.8E−07	0.005	0.014
	rs149063695_G	17:45658911	4.8	9.6E−05	2.2	0.013	4.2	0.029	3.2	2.9E−07	0.005	0.015
	rs568360281_T	17:46054408	10.4	8.5E−05	3.3	6.7E−03	6.2	0.018	5.7	5.0E−08	0.002	0.012
	rs569885052_T	17:46185108	8.3	5.3E−05	6.0	3.3E−03	8.8	0.041	7.5	5.3E−08	0.002	0.011
	**rs559612720_C**	17:46238735	13.2	1.6E−05	4.6	1.5E−03	11.4	0.021	8.1	2.1E−09	0.002	0.014
	rs537343973_A	17:46711519	5.1	3.4E-08	1.6	0.046	2.5	0.020	2.6	1.0E−09	0.010	0.026
	rs549975035_T	17:46732251	5.1	3.4E-08	1.6	0.046	2.3	0.039	2.7	1.2E−09	0.010	0.025
	rs576161544_G	17:46763849	5.4	4.5E-08	1.9	8.5E-03	2.9	0.011	3.1	5.0E−11	0.008	0.025
	**rs138213197_T**	17:46805705	6.9	2.2E−07	3.4	2.9E−03	8.7	4.2E−03	5.6	2.4E−11	0.003	0.018
	rs138467395_C	17:46860777	3.4	1.2E−06	1.7	0.049	2.6	0.026	2.5	2.1E−08	0.009	0.023

Linkage disequilibrium (LD) is depicted for controls (greyscale, *R*^2^ = 1 in black and *R*^2^ = 0 in white). Results for each study population are presented for models regressing cancer status against a given variant with adjustment for the first ten principal components of genetic ancestry. Bold font designates sentinels best detecting independent risk signals. The *HOXB13* G84E variant is rs138213197.

Pairwise LD patterns (illustrated in Table 2) suggested the potential presence of risk signals separate from the known *HOXB13* G84E. We investigated this by two complimentary approaches. We first analyzed the effect of each Table 2 variant conditioned upon G84E. Each Table 2 variant centromeric to and including rs559612720 remained significant in pairwise multivariable logistic regression models for combined study subjects that were conditioned on G84E (see Supplementary Table S2). These analyses suggested the presence of one or more risk effects separate from that of G84E. We then sought Table 2 variants best detecting the risk signal of each LD bin, and those detecting independent risk signals across LD bins. For this we used the systematic RISSc algorithm^19^ and data of combined subjects. This identified the sentinels rs559612720 and *HOXB13* G84E, each remaining significant in a mutually adjusted model (discussed below). The two sentinels are in partial linkage disequilibrium (*R*^2^ = 0.47). Relaxation of RISSc algorithm *P-*value marking threshold from 0.01 to 0.05 further identified rs559798379 as a third potential sentinel, although it had not reached genome-wide significance. Under an alternative approach of forward stepwise regression with a *P* < 0.01 threshold, rs559612720 and *HOXB13* G84E were also selected as sentinels; relaxation to a *P* < 0.05 threshold again resulted in the additional selection of rs559798379 as a third potential sentinel. The association of rs559612720 and *HOXB13* G84E with prostate cancer replicated across independent study populations, with genome-wide significance among combined subjects, and retaining significance when adjusted for each other.

Among combined cases of Table 1, rank sum tests of association with age of diagnosis yielded *P* = 0.012 for sentinel rs559612720 and *P* = 1.4E−7 for rs138213197 (each with relative excess in younger cases). Analysis of the effects of these genotypes upon age of diagnosis that were adjusted for genetic ancestry revealed that mean age of diagnosis which was 2.04 years younger for rs559612720_C carriers (*P* = 0.008) and 3.73 years younger for rs138213197_T carriers (*P* = 2.2E−8). With additional adjustment for study site, only the latter retained significance. A dichotomized case-case logistic regression model adjusted for genetic ancestry that compared combined cases diagnosed under age 60 to those diagnosed at age 60 and above yielded *P* = 4.5E−4 for rs559612720 and *P* = 1.3E−7 for rs138213197, with relative excess of the risk alleles in the younger case group. Logistic regression models that compared all combined cases diagnosed under age 60 to controls yielded odds ratios for prostate cancer of 14.0 (*P* = 2.6E−12) for rs559612720 and 10.6 (*P* = 4.1E−17) for rs138213197; among cases diagnosed at age 60 or older, these odds ratios were 6.5 (*P* = 1.4E−7) and 4.2 (*P* = 7.9E−8), respectively. The pattern of greater risk among cases diagnosed at a younger age was also observed for cases of each study population separately, as well as for the subset of cases meeting criteria for hereditary prostate cancer (Supplementary Table S2).

Categorical severity data was available only for ICPCG and NFPCS cases. Pairwise case-case comparisons of categorical severity case groups (aggressive, moderate, insignificant) were not significant for either sentinel, although a relative excess of risk alleles was observed in the more-aggressive group in each comparison. In case–control comparisons that modeled each sentinel individually with adjustment for genetic ancestry, risk of prostate cancer among all combined cases of the ICPCG and NFPCS for rs559612720 was OR = 13.1 (*P* = 6.4E−7), and for rs138213197 was OR = 7.6 (*P* = 1.3E−9). Comparison of insignificant severity cases to controls yielded OR = 8.1 (*P* = 4.0E−3) and OR = 6.6 (*P* = 4.2E−4) for the respective sentinels. Comparison of moderate severity cases to controls yielded OR = 11.4 (*P* = 1.4E−5) and OR = 6.1 (*P* = 9.1E−7) for the respective sentinels. In contrast, comparison of aggressive cases to controls yielded OR = 15.6 (*P* = 1.8E−7) and OR = 9.1 (*P* = 1.5E−10) for the respective sentinels. With further stratification to compare aggressive cases that were also diagnosed under age 60 to controls, these risks were OR = 21.2 (*P* = 7.9E−8) and OR = 10.8 (*P* = 4.4E−10), respectively. Models under mutual adjustment are presented below. Risk effects were relatively stronger among cases with a positive family history, with an early age of diagnosis, and with aggressive disease (Supplementary Tables S1 and S2).

### Multivariable modeling of sentinel risk effects

A mutually adjusted model of risk conveyed when either sentinel was inherited alone, or when inherited together, is presented in Table 3. Among subjects from all studies combined, mutually-adjusted risk was 3.6-fold for rs559612720_C (*P*_*adj*_ = 1.1E−3) and 3.1-fold for rs138213197_T (*P*_*adj*_ = 1.3E−4) if either were inherited alone, and 11.3-fold (*P*_*adj*_ = 6.2E−11) if inherited together. These results were not meaningfully altered with adjustment for study site (Supplementary Table S3). Among risk allele carriers, 13% inherited rs559612720_C alone, 37% inherited rs138213197_T alone, and 50% inherited both. The results of logistic regression models of phased subject haplotypes were similar: rs559612720_C–rs138213197_C OR = 3.2 (*P* = 0.032); rs559612720_T-rs138213197_T OR = 3.0 (*P* = 5.6E−4); and rs559612720_C-rs138213197_T OR = 11.9 (*P* = 8.2E−8). These data were consistent with an additive inheritance model, where joint inheritance of both alleles conveyed multiplicative rather than synergistic risk (interaction term *P* > 0.05). rs559612720_C had a greater effect size than rs138213197_T when inherited alone, but was also less frequent and so was accompanied by less prominent significance. Constrained statistical power limited the ability of each independent study population to separately measure risk effects of the two sentinels, although they each shared the pattern of greater risk when both sentinels were inherited together than when either was inherited alone. Results for combined ICPCG and NFPCS hereditary prostate cancer subjects are presented in Table 3. Stratification revealed that risk effects were stronger among cases with hereditary prostate cancer, with an early age of diagnosis, and with aggressive disease. The most extreme measured risk was among men who carried both sentinels and met all three criteria (OR_*adj*_ = 27.7, *P*_*adj*_ = 2.1E−8).

**Table 3:** HOXB locus sentinel risk effects, modeled under mutual adjustment.

Sentinel		NFPCS + ICPCG + PLCO Combined case vs control			NFPCS + ICPCG HPC^b^ case vs control			NFPCS + ICPCG HPC case vs control Age of Dx < 60 years			NFPCS + ICPCG HPC case vs control^c^ Aggressive			NFPCS + ICPCG HPC case vs control Age Dx < 60 years & aggressive		
rs559612720	^a^rs138213197 (*HOXB13* G84E)															
		OR_adj_	95% CI	*P*_*adj*_	OR_adj_	95% CI	*P*_*adj*_	OR_adj_	95% CI	*P*_*adj*_	OR_adj_	95% CI	*P*_*adj*_	OR_adj_	95% CI	*P*_*adj*_
		3.6	1.7 – 7.9	1.1E−03	4.5	1.4 – 14.1	0.010	4.6	1.3 – 15.7	0.016	4.9	1.5 – 15.9	8.5E−03	5.1	1.3 – 20.5	0.021
		3.1	1.7 – 5.5	1.3E−04	4.1	1.9 – 8.7	2.2E−04	5.1	2.3 – 11.5	8.5E−05	4.8	2.2 – 10.6	7.5E−05	5.4	2.1 – 14.0	4.8E−04
		11.3	5.4 – 23.3	6.2E−11	18.4	6.5 – 52.6	5.3E−08	23.3	7.8 – 69.6	1.6E−08	23.6	7.9 – 70.4	1.3E−08	27.7	8.7 – 88.6	2.1E−08

^a^*HOXB13* G84E.

^b^Hereditary prostate cancer cases are those with a family history of ≥ 3 total affected men; each evaluated case is from an unrelated pedigree.

^c^Aggressive is defined: ≥ pT3, or N1, or M1, or Gleason ≥ 8, or PSA ≥ 20 ng/ml at diagnosis, or lethal prostate cancer. The denominator of odds ratios represents carriers of only non-risk alleles of both sentinels. The numerator of these odds ratios represents carriers of either, or both risk alleles (bolded). HPC, hereditary prostate cancer. Dx, diagnosis.

### Confirmatory evaluation of sentinel genotype

Genotype inference by imputation from dense array data and the Haplotype Reference Consortium (HRC) whole genome sequence would have been subject to some error rate, accentuated for rarer variants even in data passing quality control metrics. Importantly, imputation was done blinded to affection status. While array data was the basis for imputation of each study population, NFPCS subjects were also directly genotyped by custom assays for the seven variants at genome-wide significance. Comparison of directly-assayed vs imputed genotypes revealed a discrepancy rate of 0.2%: of 10,703 genotypes, 20 genotypes had been imputed as major allele homozygotes rather than heterozygotes, and 2 genotypes had been imputed as heterozygote rather than major allele homozygote.

A sentinel risk allele is inherited in the context of an ancestral haplotype defined by surrounding alleles. The major (non-risk) allele of a sentinel may be observed on a haplotype that would otherwise typically carry the risk allele, and could indicate error. Alternatively, this may characterize the background on which the risk allele arose. Supplementary Fig. S1 illustrates aligned haplotypes for all subjects carrying at least one of the risk alleles of Table 2. The figure depicts 3538 variants distinguishing haplotypes of 388 subjects that carried risk alleles; 2211 of these variants had been genotyped in one or more of the study populations. Each variant is represented as a vertical column ordered by genomic position, while each horizontal row depicts a subject risk allele-carrying haplotype. Subjects sharing an extended haplotype of regional risk alleles (in red) are visible across the central horizontal portion of the figure. Immediately above and below haplotypes within the blue box are carriers of either sentinel risk allele alone, many of which are recombinant haplotypes. Also visible are subjects who share the haplotype on which both sentinel risk alleles can be carried, and yet do not carry both risk alleles (potential imputation error). However, this latter category included NFPCS subjects for whom sentinel genotypes were experimentally confirmed; among them were both alternatives of carriage of either risk allele alone. Overall, 71 of 98 subjects who carried only one of the two sentinel risk alleles were supported by recombinant haplotypes and/or directly-assayed sentinel genotypes. Most were not ascribable to sentinel variant imputation errors. With omission of the remaining 27 subjects, the result of a multivariable model of combined subjects was not substantively altered.

## Discussion

Our results support the existence of previously unknown HOXB locus genetic variation carrying strong risk of prostate cancer among men of European ancestry. Other than *HOXB13* G84E, each of the variants presented in Table 2 had not previously been reported. These associations were concordantly observed in each of three independent study populations, and seven of them reached genome-wide significance. Linkage disequilibrium patterns suggested the presence of novel prostate cancer risk signals, independent of *HOXB13* G84E. Multivariable models identified rs559612720 and rs138213197 (*HOXB13* G84E) as sentinels, as well as a third potential sentinel (rs559798379) that approached but did not reach genome-wide significance. The novel sentinel rs559612720 resides within intron 11 of src kinase-associated phosphoprotein 1 (*SKAP1*), 567 kb centromeric to the G84E sentinel within *HOXB13*. The interval between them encompasses multiple HOXB gene family members, illustrated in Fig. 1. Both variants are at conserved positions (GERP scores 4.39 and 4.73, respectively) and are predicted to have potentially deleterious functional effects (CADD scores 18.9 and 27.4, respectively). They are in partial linkage disequilibrium (*R*^2^ = 0.47). Among risk allele carriers, 13% inherited rs559612720_C alone, 37% inherited rs138213197_T alone, and 50% inherited both. This pattern was observed in experimental as well as imputed genotype data. Risk of prostate cancer among men inheriting both sentinel risk alleles was the product of risk attributable to each separately, consistent with a multiplicative model of risk driven by two sentinels. This observation could also be consistent with an alternative model of an undetected causal mutation that is partially correlated with both sentinels. The data were not consistent with a model of *HOXB13* G84E as a lone risk variant within the HOXB locus. Current NCCN Guidelines for familial cancer care (and commercial testing panels) encompass the G84E mutation (rs138213197_T), which in study data would underestimate risk in carriers of both sentinels as well as risk in carriers of sentinel rs559612720_C alone. These risks were greatest for early-onset and aggressive disease (corroborating facets previously known for G84E^2,21^).

*SKAP1* is widely expressed in tissues, including prostate, with principal expression in whole blood where it is selectively expressed by T cells, macrophages, and mast cells^22–24^. SKAP1 (also known as SKAP55) is an adapter protein of the T-cell receptor at the interface of CD8 cytotoxic T lymphocytes and tumor cells, functioning in adhesion and anti-tumor immune response^25^. SKAP1 expression has been observed to be correlated with cytotoxic T cell PD-1 expression, suggesting a role in tumor tolerance^25^. A genetic variant within *SKAP1* has previously been found to be associated with prostate cancer specific mortality^26^. Variants within *SKAP1* have also been identified in GWAS of endometrial and ovarian cancer^27–31^; methylation of this gene is also correlated with ovarian cancer risk^32^. In The Cancer Genome Atlas (TCGA) prostate cancer data, reduced *SKAP1* expression is associated with a younger age of diagnosis, and expression is reduced in prostate adenocarcinoma relative to normal prostate. Reduced *SKAP1* expression is also associated with worse survival in TCGA bladder and breast cancer, with a similar but insignificant trend in prostate and numerous additional cancers^23^. However, the risk signal that sentinel rs559612720 detects could mediate its effect through a gene other than the *SKAP1* gene in which it resides, for example by distal regulation of *HOXB13* or another gene. A third potential risk signal, detected by rs559798379, was found within the LD block centromeric to that harboring *SKAP1*. While that sentinel could also detect some distal regulatory function, it may alternatively indicate the involvement of a separate disease gene. *CDC27* of that block, for example, is notable for recurrently observed tumor somatic mutations^33–35^ and functions in the anaphase-promoting complex with a role in mitotic segregation errors^36^. These sentinels identify heritable prostate cancer risk that is distinct from that attributable to *HOXB13* G84E, but mechanistic studies are required to advance from clinical association to causality.

Genetic enrichment expected of the familial cases of the ICPCG and NFPCS would improve power to detect rarer and stronger effect variants (motivating the familial case–control study design), and would yield effect sizes reflecting the greater risk among men with a family history of prostate cancer. All twelve risk variants of Table 2 carried notable risk effects in the independent familial case–control populations of both the NFPCS and ICPCG. The PLCO case–control data set further extends these observations to a study population that was unselected for family history. Effect sizes of these variants were uniformly greater in NFPCS and ICPCG hereditary prostate cancer cases than in PLCO cases (Supplementary Table S2); even so, measured effects within the PLCO were considerably greater than those of typical GWAS SNPs. PLCO cases were from a prospective screening trial, with measured effect sizes reflecting risk among men unselected for a family history. This would make the PLCO less likely than a familial study population to detect rare variants of strong effect. It is noteworthy that the variants of Table 2 replicated in the PLCO despite this heterogeneity.

Study differences also present potential limitations. The ICPCG data set aggregated hereditary prostate cancer cases from multiple separate global study populations (Australia, Finland, France, Germany, UK, and US). The NFPCS and PLCO investigated US cases. Given the geographic diversity across study sites and potential for substructure, all analyses were adjusted for genetic ancestry. A subset of ICPCG hereditary prostate cancer cases were selected for more aggressive disease, whereas NFPCS and PLCO cases were not. An early age of diagnosis is a recognized clinical facet of hereditary prostate cancer, although aggressiveness is not^12^. Subsets of cases of the ICPCG and NFPCS were selected based upon an early age of diagnosis. PLCO cases had a later mean age at diagnosis (69 yr, vs 60 yr for ICPCG, 57 yr for the NFPCS, and 66 yr as the US and UK national means). Ages did not accompany ICPCG control data (and were not used in prior analyses^17^), however, adjustment for age among NFPCS and PLCO subjects (Supplementary Table S1) did not meaningfully alter results.

The G84E germline mutation of *HOXB13* is pertinent for hereditary cancer care^37^ and is clinically evaluated within commercial panels. Our study observed that the prostate cancer risk that this locus carries can significantly exceed that which may be appreciated by testing *HOXB13* G84E alone. Among families with three or more affected men, risk measured for the sentinel pair had effect sizes analogous to those known for breast cancer predisposition by pathogenic variants of *BRCA1* and *BRCA2*^38^. Our results indicate that evaluation of an additional locus sentinel, rs559612720 in *SKAP1,* would more fully capture prostate cancer risk of this locus than rs138213197 in *HOXB13* alone. The multiplicative effects observed could meaningfully impact clinical assessment of individual patient risk, particularly risk of early onset, aggressive prostate cancer. Study patients who did not carry the *HOXB13* variant but carried the *SKAP1* variant had 3.6-fold elevated risk for prostate cancer, not baseline risk as might otherwise be interpreted by a negative clinical *HOXB13* test. Moreover, a man who had inherited both *HOXB13* G84E and the *SKAP1* sentinel could have considerably greater risk of early onset, aggressive prostate cancer than might be appreciated by knowledge of G84E carriage alone. Epidemiologic studies of prostate cancer could analogously be impacted by an incomplete ability of *HOXB13* G84E to fully capture locus risk. Heterogeneity of measured *HOXB13* G84E mutation risk across distinct studies might result as a function of differing carrier proportions of adjacent and untested locus sentinels. This is likely to be correlated with study population characteristics such as family history, age of diagnosis, and pathologic severity^6^, and should be considered with further studies.

## Methods

### Study populations

Subject counts and characteristics of each study population are summarized in Table 1.

### International Consortium for Prostate Cancer Genetics Study (ICPCG)

Data of the ICPCG GWAS of Familial Prostate Cancer was from dbGaP, accession phs000733.v1.p1. Case and control selection criteria are previously published^17^ and detailed in dbGaP meta-data. The data set encompasses 2505 analyzed unrelated hereditary prostate cancer cases aggregated from 12 studies conducted at the following sites: Cancer Council Victoria (Australia), the Center for Research on Prostatic Diseases (France), the Fred Hutchinson Cancer Research Center (US), the Institute of Cancer Research (UK), Johns Hopkins University (US), Louisiana State University (US), the Mayo Clinic (US), Northwestern University (US), Tampere University (Finland), the University of Michigan (US), the University of Ulm (Germany), and the University of Utah (US). These sites each employed uniform criteria to ascertain hereditary prostate cancer pedigrees^11^. One case was selected from each previously ascertained pedigree with a total of ≥ 3 affected male relatives. Within a given pedigree, a case with more aggressive disease or with an early age of diagnosis was preferentially selected for genotyping. Case phenotypic data include age at diagnosis and categorical severity. Nine sites also contributed 1383 unrelated male controls without a cancer diagnosis. Age of diagnosis for each case was recorded, and control ages were of similar distribution though not individually recorded in the data set^17^. All subjects were of self-reported European ancestry. Genotype data was generated with the Omni5Exome array with quality control described in dbGaP metadata.

Cases were categorized into severity groups that mirror criteria of NCCN Guidelines.

Aggressive: extra-prostatic stage at diagnosis (≥ T3, N1, or M1), or Gleason ≥ 8 (poorly differentiated), or PSA ≥ 20 ng/ml at diagnosis, or lethal prostate cancer.

Insignificant: stage T1 or in only one lobe (T2a) if prostatectomy done, and no evidence of extra-prostatic disease, and Gleason ≤ 6 (not moderately or poorly differentiated), and PSA ≤ 4 ng/ml at diagnosis, and if deceased did not die of prostate cancer.

Moderate: cases not meeting either aggressive or insignificant criteria.

### Nashville familial prostate cancer study (NFPCS)

The NFPCS is a case–control study, described in prior publications^1,18,19,39–41^ and briefly summarized here. The study was conducted in accordance with Institutional Review Board oversight with written informed consent. Subjects were recruited in the course of standard care at Vanderbilt University Medical Center and Veterans Administration Hospital in Nashville, TN between 2003 and 2009. Subjects included incident cases undergoing treatment for prostate cancer, and incident controls undergoing routine preventative screening. Case inclusion criteria required each proband to have a family history of one or more 1st or 2nd degree relatives with prostate cancer (total of ≥ 2 affected male relatives in the family). Among case probands with only one affected relative, only the subset of probands that were diagnosed at an age under the US national mean of 66 years were evaluated. In contrast, all case probands with two or more 1st or 2nd degree affected family members (total of ≥ 3 affected male relatives in the family, meeting hereditary prostate cancer criteria) were evaluated, irrespective of age at diagnosis. Controls were required to have a negative personal and family history of prostate cancer, no known abnormal digital rectal examination, no prior prostate biopsy, a screening prostate specific antigen (PSA) below 4 ng/ml (93% were below 3 ng/ml), and all prior known PSA levels also below this level. The age profile of controls (Table 1) was slightly older than that of cases, which is conservative (genotype and phenotype would be unchanged if a given control had been recruited at the same age as a younger case). A series of cases of early age of diagnosis but without a family history of prostate cancer was also separately recruited for study. The subset of NFPCS subjects of self-reported European ancestry was included in this investigation, enabling comparison to the other study populations.

Radical prostatectomy was the treatment modality for 97% of case subjects, providing pathologic stage and grade. DNA prepared from whole blood was genotyped by Illumina Multi-Ethnic Genotyping Array (MEGAex). Blinded duplicate study samples and HapMap trios were included for quality control. Array data was processed by GenomeStudio with de novo clustering and quality control using the pipeline of the Vanderbilt biobank^19,42^. Orthogonal genotype assays were completed by restriction fragment length polymorphism assay (rs576161544), by TaqMan (rs138213197), or by single-nucleotide primer extension with detection by fluorescence polarization (rs568360281, rs559612720, rs537343973, rs549975035, and rs138467395)^43^. All NFPCS subjects were genotyped for each custom assay.

### Prostate, lung, colorectal, and ovarian cancer screening trial

Data of the PLCO Trial^44^ were from the Prostate Cancer GWAS for Uncommon Susceptibility Loci (PEGASUS) GWAS^20^, dbGaP phs000882.v1.p1. The PLCO is a large US population-based trial to determine the effects of screening on cancer-related mortality in men aged 55 to 74. Recruitment began in 1993 and closed in 2001. Men with a prior history of prostate, lung, or colorectal cancer were excluded. 38,340 men were screened by PSA and digital rectal exam for 6 years after recruitment and followed for at least 7 years for prostate cancer outcomes, for comparison to 38,345 who had usual medical care. Screening was completed in 2006 with continued data collection through 2015. The data set encompasses 2840 controls and 4,544 incident prostate cancer cases of European ancestry. Subjects were unselected for family history of prostate cancer. Phenotype data accompanying cases includes age at diagnosis and Gleason grade (the latter insufficient for severity categorization by criteria given above). Subjects were genotyped using the Illumina HumanOmni2.5 array with quality control described in dbGaP metadata and in reference^20^.

### Whole genome sequence-based imputation

The University of Michigan Imputation Server pipeline was employed for genotype imputation of each study population. Array-generated genotypes were used as the basis for imputation against reference whole genome sequence of 32,488 subjects of the Haplotype Reference Consortium r1.1 2016^45^. Only informative bi-allelic SNPs with a required minimum genotype completion rate of 98% and subject completion rate of 98% were used as the basis for imputation. Because rarer variation is pertinent in familial prostate cancer, it was included rather than filtered. Phasing employed Eagle v2.3 with imputation using Minimac 3^46^. The most probable genotypes for imputed variants of *R*^2^ ≥ 0.75 were retained, while those of lower quality were filtered. This yielded 5,517 informative variants within the genomic interval between 17:45,416,600 and 17:46,860,777 (GRCh37/hg19) with complete data in ICPCG, NFPCS, and PLCO study subjects. Hardy–Weinberg equilibrium (HWE) filters were not employed because pertinent mutations could impact population fitness. Genotype data was derived independently of trait status; genotype error as a source of disequilibrium would bias toward a null disease association. Genetic variants of Table 2 were each of HWE *P* ≥ 0.05 within each study population.

### Genetic ancestry

We confirmed subject genetic independence (proportion identity by descent ($\widehat{\pi }$ < 0.05)) within and across study populations. Subjects of each study population were of self-reported European ancestry, and in past work had been imputed against the 1000 Genomes Phase 3 reference with confirmation of corresponding European genetic ancestry: the ICPCG used the principal components-based SNPRelate program^17,47,48^; the NFPCS used the cluster-based STRUCTURE program^19,49^; and the PLCO (PEGASUS) employed the struct.admix module of glu-genetics^20^. We conducted principal components analysis of the current data to enable statistical adjustment for genetic ancestry, given potential for differences across the distinct global recruitment sites. We used FlashPCA2.0 and pruned, post-imputation genome-wide genotype data to calculate principal components (40,964 variants of MAF ≥ 0.01, HWE *P* ≥ 5E−5, genotype missingness ≤ 0.001, and variant independence using a pairwise *R*^2^ cutoff of ≤ 0.02)^50^. The principal components analysis was performed in the full sample of all subjects of the combined studies.

### Statistical analyses

Unconditional logistic regression models were employed to identify associations between genetic variants and prostate cancer, for each individual study population. These models were additive on the logit scale (additive genetic models) and adjusted for the first ten principal components of genetic ancestry. Significance was assessed using Wald tests. An association was considered nominally significant with a two-sided *P* ≤ 0.05. In order to assess overall significance and effect size, variants were further evaluated in combined study data by models adjusted for the first ten principal components of genetic ancestry (adjusting for potential substructure across study sites). To assess the effect of age as a potential confounder in the NFPCS and PLCO, we additionally evaluated models adjusted for genetic ancestry as well as age (control ages did not accompany ICPCG data, and were not used in prior analyses^17^). Adjusting for age did not meaningfully alter results (Supplementary Table S1). Adjustment for genetic ancestry as well as study site (nine separate ICPCG sites, NFPCS, and PLCO) also did not meaningfully alter results (Supplementary Tables S1, S2, and S3). Associations were considered to be genome-wide significant by the convention^51^ of *P* ≤ 5 × 10^–8^. STATA v16 and PLINK v1.9 were used for statistical analyses^52^. Haplotypes of Supplementary Fig. S1 were generated by joint phasing of combined subjects using Beagle v5.1^53^.

We employed the RISSc algorithm to identify sentinel SNPs among the variants at genome-wide significance in combined study subjects^19^. This algorithm identifies variants that optimally detect the risk signal of a given linkage disequilibrium (LD) bin, and those which detect independent risk signals across LD bins under mutual adjustment. The algorithm can improve upon an alternative forward/backward stepwise regression approach^19^. Our analyses employed a stepwise LD bin increment of *R*^2^ = 0.025 and marking threshold of *P* ≤ 0.01. Selected sentinels were analyzed among subjects of the combined studies in multivariable models adjusted for the first ten principal components of genetic ancestry. Further multivariable models of identified sentinels evaluated stratified case groups: those meeting hereditary prostate cancer criteria, diagnosed prior to age 60, or meeting criteria for aggressive pathology. For haplotypic analysis of identified sentinels, diplotypes were resolved using Phase v2.1^54^. Haplotypes were tested for association with prostate cancer under additive logistic regression models adjusted for the first ten principal components of genetic ancestry.

The Wilcoxon rank-sum test was used to assess association between genotype and age of diagnosis among cases of the combined studies. Case-only linear regression models that were adjusted for principal components of genetic ancestry were used to assess the effect of genotype on age of diagnosis. We also investigated potential association of variants with prostate cancer severity for NFPCS and ICPCG cases, each categorized into severity groups using the criteria given above. PLCO cases were accompanied by insufficient phenotype data to enable categorization. Case severity groups were evaluated in dichotomized comparisons (aggressive vs insignificant, moderate vs insignificant, and aggressive vs moderate) by logistic regression models adjusted for the first ten principal components of genetic ancestry. Aggressive cases and non-aggressive (insignificant plus moderate) cases were also separately compared to controls by logistic regression models adjusted for the first ten principal components of genetic ancestry.

## Supplementary Information

Supplementary Information 1.

Supplementary Information 2.
