# Supplementary material for: Prostate cancer risk variants of the HOXB genetic locus
Source: Sci Rep. 2021 May 31;11:11385. doi: 10.1038/s41598-021-89399-7 (PMC8167119; doi:10.1038/s41598-021-89399-7)
Supplement: Supplementary file 1 — Supplementary Information 1. [file 41598_2021_89399_MOESM1_ESM.pdf]

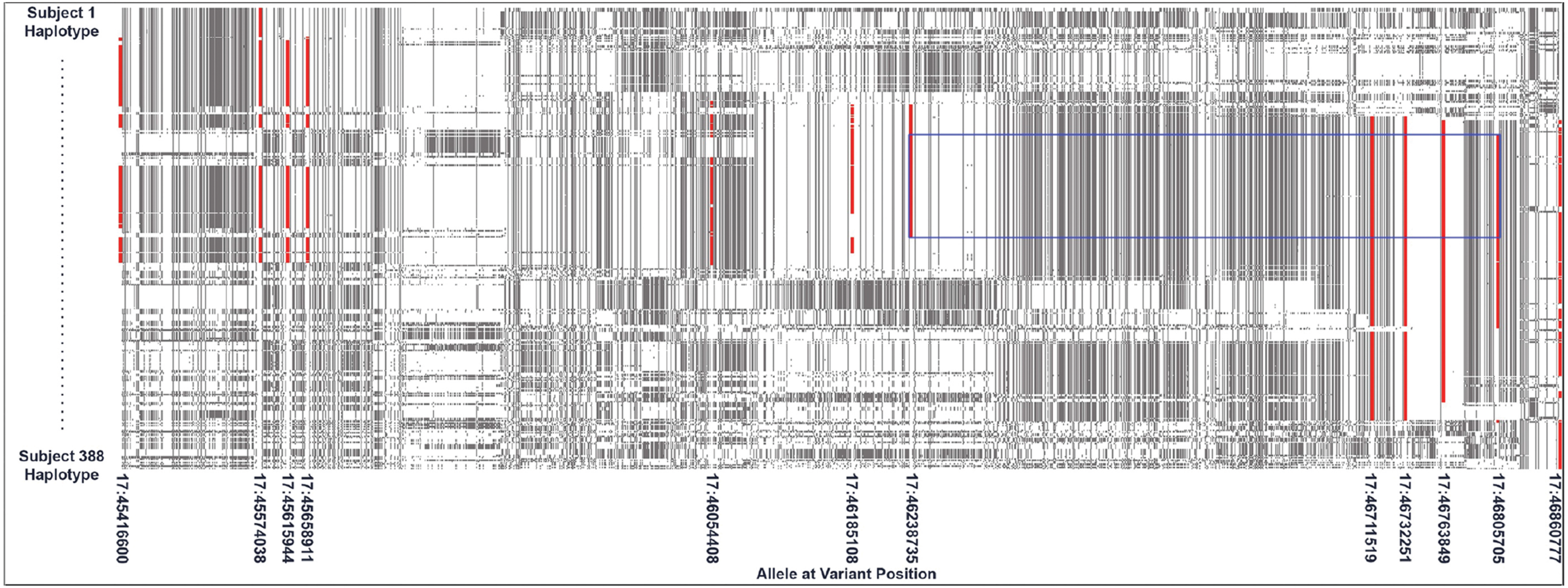


**Supplementary Figure S1**. **Haplotypes of prostate cancer risk allele carriers at the 17q21 HOXB locus.** The subset of genetic variants that distinguish haplotypes carrying identified prostate cancer risk alleles are presented ordinally along the X-axis (3,538 variants of chr17:45,416,600-46,860,777). Rows of the Y-axis present risk-allele carrying haplotypes of 388 study subjects. Minor alleles are depicted with black shading and major alleles are in white shading. Risk alleles of Table 2 are highlighted in red. Subjects carrying a haplotype harboring both sentinel risk alleles are denoted by the blue box (red sentinel alleles are at right and left box boundaries). Just above or below the box are subjects sharing the haplotype but carrying only one of the two sentinel risk alleles; further above and below are haplotypes that diverge, with past recombination events.
